# A High-Precision Magnetic-Assisted Heading Angle Calculation Method Based on a 1D Convolutional Neural Network (CNN) in a Complicated Magnetic Environment

**DOI:** 10.3390/mi11070642

**Published:** 2020-06-29

**Authors:** Guanghui Hu, Hong Wan, Xinxin Li

**Affiliations:** 1 State Key Laboratory of Transducer Technology, Shanghai Institute of Microsystem and Information Technology, Chinese Academy of Sciences, Shanghai 200050, China; guanghui_hu@mail.sim.ac.cn; 2 School of Microelectronics, University of Chinese Academy of Sciences, Beijing 100049, China; 3 Vtran Tech (Chang Zhou) Co., Ltd., Shanghai 200135, China

**Keywords:** magnetic-assisted, one-dimensional convolutional neural network (1D CNN), magnetic anomaly detection, pedestrian inertial navigation

## Abstract

Due to its widespread presence and independence from artificial signals, the application of geomagnetic field information in indoor pedestrian navigation systems has attracted extensive attention from researchers. However, for indoors environments, geomagnetic field signals can be severely disturbed by the complicated magnetic, leading to reduced positioning accuracy of magnetic-assisted navigation systems. Therefore, there is an urgent need for methods which screen out undisturbed geomagnetic field data for realizing the high accuracy pedestrian inertial navigation indoors. In this paper, we propose an algorithm based on a one-dimensional convolutional neural network (1D CNN) to screen magnetic field data. By encoding the magnetic data within a certain time window to a time series, a 1D CNN with two convolutional layers is designed to extract data features. In order to avoid errors arising from artificial labels, the feature vectors will be clustered in the feature space to classify the magnetic data using unsupervised methods. Our experimental results show that this method can distinguish the geomagnetic field data from indoors disturbed magnetic data well and further significantly improve the calculation accuracy of the heading angle. Our work provides a possible technical path for the realization of high-precision indoor pedestrian navigation systems.

## 1. Introduction

The application of global positioning systems (GPS) in the field of navigation has resulted in greater convenience for human society. However, in some scenarios, such as rural areas, indoors, and during field rescue, navigation based on satellite positioning technology is far from being sufficient in meeting the requirements. At the same time, with the development of microelectronics manufacturing and algorithm theory, researchers have begun to study new technological solutions to meet diverse navigation requirements in the case of GPS failure [1,2,3,4]. Hence, the emergence of various high-precision indoor navigation technologies, including wireless signals [5], images [6], maps, etc. Among them, indoor pedestrian inertial navigation routes, based on inertial sensors, have attracted a lot of attention due to them being autonomous and not susceptible to external jamming [7,8,9]. Additionally, inertial sensors are becoming increasingly cheaper and more widely used in smart devices, which could support the use of pedestrian inertial navigation through the development of micro-electro-mechanical systems (MEMS).

In order to take advantage of MEMS-based inertial autonomous navigation, Suprem et al. [10] adopted a method to correct missing GPS routes by integrating data from inertial sensors in smartphones. Prateek et al. [11] used two foot-mounted inertial navigation systems (INS), one on each foot, to reduce the heading drift in pedestrian navigation by applying a range constraint based on the spatial separation between the two feet. Li et al. [12] adopted indoor environmental modeling and construction to optimize heading angle errors. Deng et al. [13] utilized PCA over the horizontal plane of acceleration signals for local walking direction extraction. Chen et al. [14] used a floor plan, and Zhuang et al. [15] used Wi-Fi to reduce the accumulated error of an inertial navigation system. The above scholars used model constraints and peripheral auxiliary equipment to reduce the heading angle accumulation error of the pedestrian inertial navigation, resulting in an increase in the cost or complexity of the system. Comparison with the above methods, the ubiquitous geomagnetic field information could be used to solve the same problem without additional cost.

Geomagnetic field signals can provide value direction information in indoor pedestrian inertial navigation due to their flexible application scenarios and high degree of integration. Magnetic-assisted inertial navigation technology is able to use geomagnetic field data without being constrained by the scene [16,17]. However, there are a large number of magnetic signal interference sources in indoor environments, which makes the indoor magnetic environment particularly complex, therefore making geomagnetic field signals extremely susceptible to interference and distortion. In this case, the direct use of raw magnetic signals will cause errors in the navigation heading angle calculation, which will affect navigation accuracy [18]. In order to reduce magnetic signal interference, researchers have proposed a statistical-based magnetic data screening method, but this requires prior assumptions to be met and performs poorly in complex magnetic environments, which limits its application scenarios [19,20].

In recent years, rapidly developing machine learning algorithms have provided researchers with effective tools for signal processing. Deep learning algorithms, Bayesian networks, etc., have been used in many applications in classification scenarios [21,22]. As a data classification method, one-dimensional convolutional neural network (1D CNN) has powerful classification performance in time series recognition and does not rely on any assumptions, enabling its use in combining feature extraction and signal classification into a learning subject in various environments [23]. In this article, we describe the development of an algorithm based on 1D CNN to screen magnetic data and verify its performance. First, we analyzed the source of the magnetic signal and then designed a 1D CNN structure based on the characteristics of the magnetic signal. In addition, the unsupervised learning neural network—which has a powerful autonomous classification function and can autonomously analyze the characteristics of the given data and classify them into different categories with a perfectly designed network structure—was introduced to optimize our network [24,25]. The use of unsupervised learning methods eliminates the need to provide the neural network with any prior labels, thus completely avoiding any interference to the network classification performance based on various artificial assumptions. This method can also solve the problem of the magnetic interference source being difficult to locate in an indoor complex magnetic environment. Through training based on a back propagation (BP) algorithm, 1D CNN can learn to recognize the difference between the signals of different modes [26]. The 1D CNN can be well trained using the collected dataset such that it can distinguish non-interfering and interfering geomagnetic field signals. Finally, we integrated the well-trained 1D CNN into a pedestrian inertial navigation system and generated a high-precision heading angle calculation algorithm. We tested this algorithm through experiments, and the results show that our algorithm can improve the accuracy of indoor navigation.

The rest of this paper is organized as follows. Section 2 introduces the characteristics of geomagnetic fields and the navigation system with a magnetic signal screening algorithm based on 1D CNN. Section 3 shows the results of our experiments and verifies the performance of our proposed navigation system. Finally, Section 4 concludes the paper and provides some future directions.

## 2. The Principle of the System

### 2.1. Geomagnetic Signal Analysis

The Earth’s magnetic field is a naturally occurring planetary phenomenon which follows the principles of dynamo theory and is created by the constant motion of Earth’s outer core, composed mostly of molten iron [27]. The geomagnetic field can be modeled as a dipole, and the geomagnetic field lines originate at the positive pole of the dipole (magnetic south) and end at the negative dipole (magnetic north). Since the geomagnetic poles do not coincide with the geographic poles, there is a declination μ between the geomagnetic heading and the geomagnetic heading. A triaxial magnetometer can be used to measure the geomagnetic field vector. With the proper transformation of the geomagnetic field components to the horizontal plane, geographic heading ψ can be estimated using Equation (1), where $H_{x}$ and $H_{y}$ are the components of the geomagnetic field in the horizontal plane.

(1) $$\psi =\arctan\left(\frac{H_{y}}{H_{x}}\right)+\mu$$

**Figure 1:**
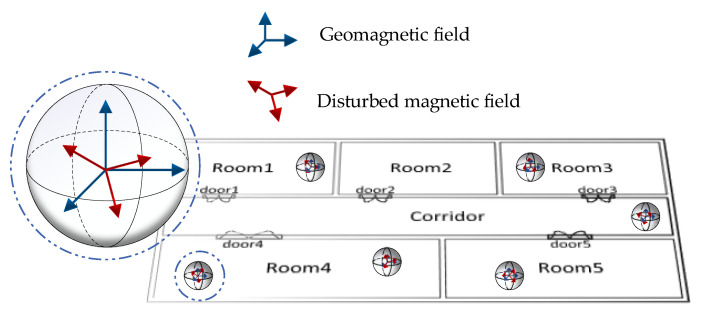
The random distribution of magnetic interference sources indoors.

**Figure 2:**
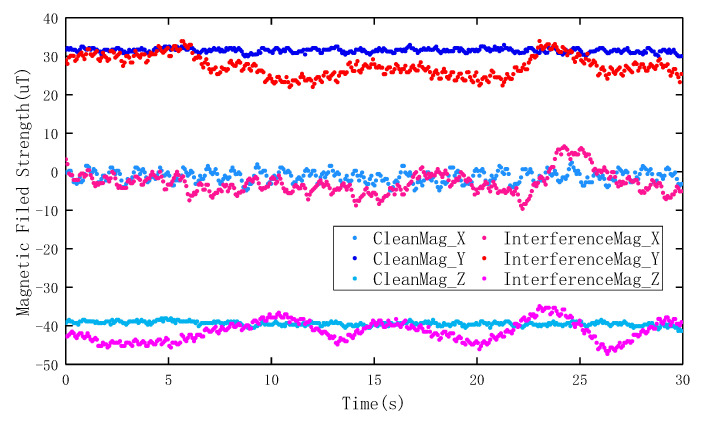
An example of the distribution of magnetic interference data in three axes.

In indoor pedestrian inertial navigation, the heading angle can be obtained using a MEMS gyroscope. The error in the heading angle calculated by the gyroscope accumulates twice over time because the gyroscope contains measurement errors. The accuracy of the heading angle obtained by geomagnetic information is not related to time. Hence, the geomagnetic field can be used to correct the accumulation error of the heading angle calculated by the gyroscope. However, there are a large number of artificial interference sources randomly distributed throughout indoor spaces, as shown in Figure 1. The geomagnetic field information measured by a magnetometer is not accurate under these conditions, and the interference magnetic data sequence will show a different value model in three axes, as shown in Figure 2. Due to magnetic anomalies, the heading angle information contained in the magnetic data can be disturbed. Correcting the heading angle by using the interference geomagnetic data will increase the systematic error. Therefore, a method is urgently needed to verify whether the geomagnetic field data are causing interference before using magnetic data to achieve a more accurate navigation performance.

### 2.2. Algorithm Structure and Process

**Figure 3:**
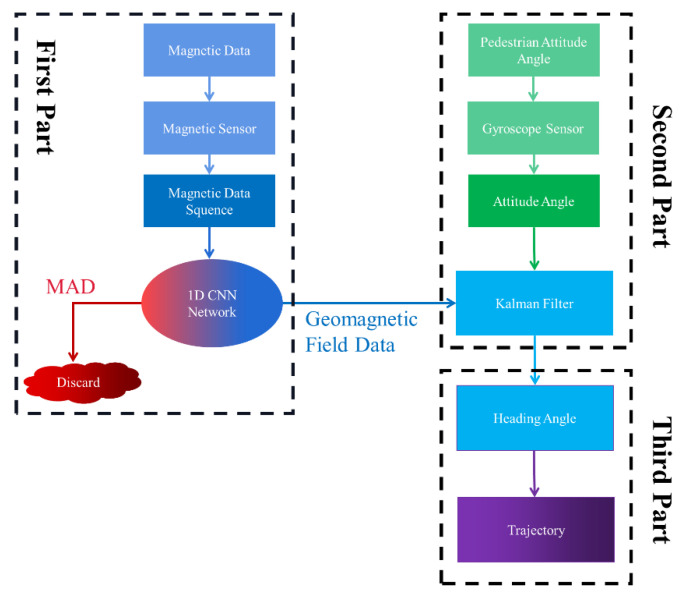
The flowchart of the heading angle calculation based on a one-dimensional convolutional neural network (1D CNN).

In order to make full use of the magnetic data information and avoid interference from using magnetic data anomalies, we designed a novel algorithm flow to calculate the heading angle. As shown in Figure 3, the algorithm can be divided into three main parts. The first part is used for the collection and screening of magnetic data. In this part, the magnetic data are collected by the magnetometer sensor and input into our algorithm. Our algorithm treats the magnetic data collected within a certain time window as a set, and we record this set as a magnetic data sequence.

The magnetic data sequence collected by the magnetometer will then be transmitted to a neural network. Once this neural network is well trained, it can be used as an indicator in the algorithm. It can determine whether the magnetic data sequence belongs to a magnetic anomaly sequence or is geomagnetic sequence data. When the neural network verifies that the received magnetic data sequence is a magnetic anomaly sequence, which contains incorrect information, our algorithm will round it down to avoid erroneous effects on the system’s heading angle calculation. When the neural network indicates that the collected magnetic data sequence is undisturbed data, the algorithm will transmit this set of data to the second part. The second part of the algorithm integrates an extended Kalman filter, which is a special form of particle filtering and can be used to correct magnetic data with a smaller computation in our devices, transmitted from the previous part, and fuse the information obtained by other inertial sensors to calculate the heading angle [28,29]. Because the instantaneous value of the inertial sensor is accurate and the first part of the algorithm can periodically provide accurate magnetic data to correct the accumulated error of the inertial sensor, the accuracy of the system’s heading angle solution will be significantly improved by this method. Following the calculation of the heading angle in the second part, in the third part, the algorithm will use this calculated heading angle to calculate the speed and acceleration of the pedestrian, thereby correcting the trajectory of the pedestrian.

### 2.3. Magnetic Anomaly Detection Based on Unsupervised Learning

**Figure 4:**
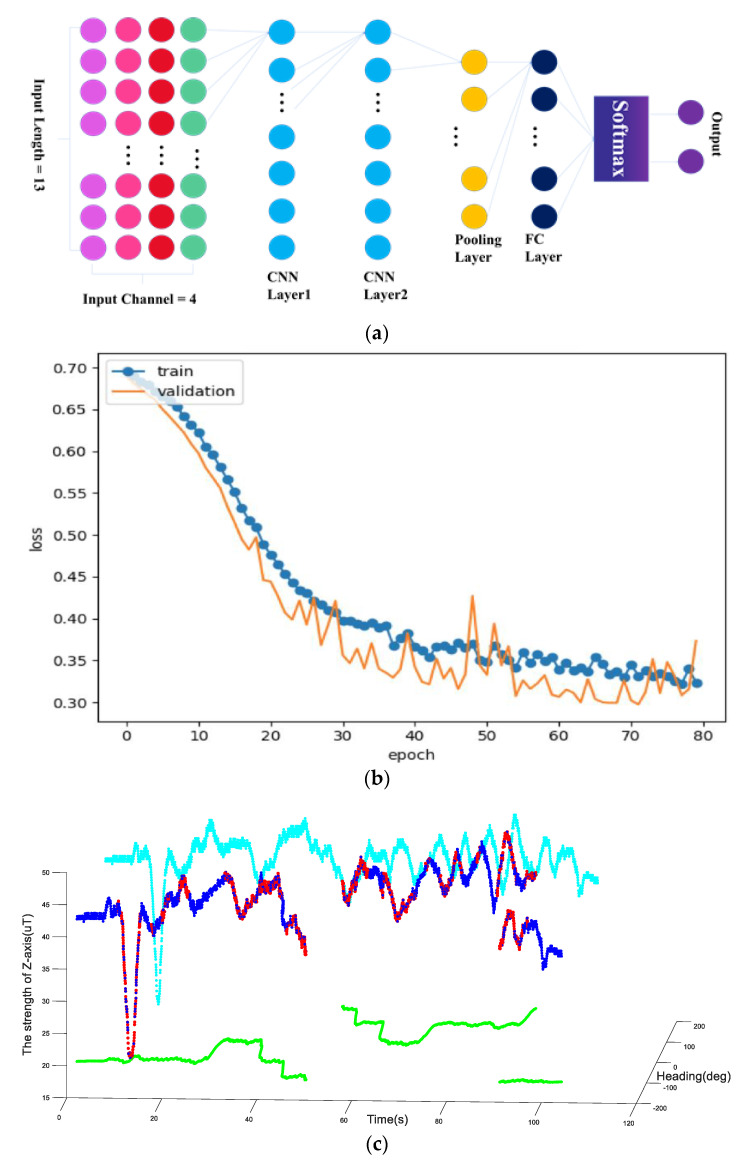
(**a**) The topological structure of the 1D CNN network. (**b**) The decrease in the loss function with training. (**c**) Magnetic data classification results based on 1D CNN.

For magnetic data screening, the second part of the algorithm includes a convolutional neural network (CNN). In order to get good screening results, we carefully defined the topology of the neural network based on the characteristics of the magnetic data. Since the collected magnetic data were three-dimensional vectors, we defined four channels consisting of three axis values and the model of the magnetic data vector for input into the neural network. We can define the dimensions of the input according to the length of the input time series. As shown in Figure 4a, the length of the time window we chose here was 13, so the dimension of the input signal was 13. After the magnetic sequence signal was transmitted into the network, we used a two-layer convolutional network to extract its features. It is obvious that the nature of the magnetic sequence depends on the relationship between adjacent data, so we can extract the data characteristics between them using a convolution operation. In the network we designed, the first layer of the network used a convolution kernel of length 4 and extracted 64 data features. In the second layer of the network, we also used a convolution kernel of length 4 to extract 64 data characteristics.

After passing through a pooling layer and a fully connected layer, we used the SoftMax function to obtain two outputs, which, respectively, indicate the network’s probability of judging whether they are in a magnetic anomaly state or geomagnetic field data state. Since the learning network is unsupervised, our training process uses a combination of forward and backward propagation algorithms. We used the K-cluster theory to define the loss function. If we note the network as function f and β represents all the parameters in the two convolutional layers and the full connection layer, the feature extracted from the input can be described as:

(2) $$v=f_{\beta }(X)$$

where X represents the magnetic data sequence:

(3) $$X=\left[\begin{array}{cccc} {\vec{M}}_{t} & {\vec{M}}_{t+1} & \cdots & {\vec{M}}_{t+12} \end{array}\right]$$

The input sequence has four channels as mentioned above, and its length is 13. The dimension of vector M is 4×1, and the dimension of X is 4×1×13. For a vector $\vec{M}$, it contains magnetic field vector and magnetic field strength. And the x-component of the vector is noted as $M_{x}$, the y-component of the vector is noted as $M_{y}$, the z-component of the vector is noted as $M_{z}$ and the strength of the vector is noted as $M_{l}$. We can rewrite it as a 4×13 matrix X:

(4) $$X=\left[\begin{array}{cccc} M_{x,t} & M_{x,t+1} & \cdots & M_{x,t+12}\\ M_{y.t} & M_{y,t+1} & \cdots & M_{y,t+12}\\ M_{z,t} & M_{z,t+1} & \cdots & M_{z,t+12}\\ M_{l,t} & M_{l,t+1} & \cdots & M_{l,t+12} \end{array}\right]$$

Therefore, the distance of the two feature vectors for the cluster is

(5) $$d_{\beta }(X,Y)=\left\|f_{\beta }(X)-f_{\beta }(Y)\right\|$$

Since the SoftMax function is used, we use w to represent the weight vector of the SoftMax layer, and we can get the probability of feature v being recognized as class i (i = 0, 1, …):

(6) $$P(i|v)=\frac{\exp(w_{i}^{T}v)}{\sum_{j=1}^{n}\exp(w_{i}^{T}v)}$$

The objective of training is maximum:

(7) $$P(\beta ,w)=\prod_{i=1}^{n}p(i|f_{\beta }(x_{i}))$$

The negative log-likelihood of the training set is:

(8) $$J(\beta ,w)=-\sum_{i=1}^{n}\log P(i|f_{\beta }(x_{i}))$$

The β represents all the parameters in the CNN network, and the w represents the weight vector of the two classes. In the stochastic gradient descended method, if we note that the learning ratio is α, the updated method of the parameters is

(9) $$\beta_{i,t}=\beta_{i,t-1}-\alpha \frac{\partial }{\beta_{i,t-1}}J(\beta ,w)$$

(10) $$w_{i,t}=w_{i,t-1}-\alpha \frac{\partial }{w_{i,t-1}}J(\beta ,w)$$

As shown in Figure 4b, by inputting a magnetic signal sequence collected from a large number of indoor environments into the network, the loss value of train data and test data (validation) will continue decrease with the training process, which implies that the network can gradually learn the difference between a normal magnetic signal and an abnormal magnetic signal in the magnetic signal sequence, and classify them. After the neural network is well trained, it can accurately complete the classification of signals. Figure 4c represents the magnetic anomaly detection results using our 1D CNN model. The bule points and red points represent the magnetic data collected by our device. The green points are the projector of the magnetic data in the XY-plane heading angle space, and the cyan points are the strength value of magnetic data in *Z*-axis. We use the blue point to indicate the geomagnetic data extracted from the magnetic data by our 1D CNN, and the red points are the interference magnetic data. As we can see, the magnetic data are divided into two groups, and we will verify the effectiveness of the separation in the following sections.

### 2.4. Geomagnetic Signal-Assisted Heading Angle Calculation

We can enter the geomagnetic field data obtained in the second part of the algorithm in Figure 3 into an extended Kalman filter (EKF) to make full use of the heading angle information contained in the geomagnetic signal and correct the error caused by the inertial navigation sensor in the system. The specific methods for the heading angle calculation are described below.

In the process of attitude angle calculation for pedestrian inertial navigation, the quaternion calculation with a simple arithmetic process can simplify the arithmetic process and improve efficiency without loss of accuracy. The relationship between the quaternion and the attitude angle can be obtained through a certain attitude angle operation in the attitude matrix, which can be represented by Equations (11) and (12), respectively. $C_{m}$ represents the attitude matrix, $\left[\begin{array}{cccc} q_{0} & q_{1} & q_{2} & q_{3} \end{array}\right]$ represents the quaternion, and the attitude angles are ψ for the heading angle, θ for the pitch angle, and ϕ for the roll angle.

(11) $$C_{b}^{n}=\left(\begin{array}{ccc} \cos\theta \cos\psi & -\cos\phi \sin\psi +\sin\phi \sin\theta \cos\psi & \sin\phi \sin\psi +\cos\phi \sin\theta \cos\psi \\ \cos\theta \sin\psi & \cos\phi \cos\psi +\sin\phi \sin\theta \sin\psi & -\sin\phi \cos\psi +\cos\phi \sin\theta \sin\psi \\ -\sin\theta & \sin\phi \cos\theta & \cos\phi \cos\theta \end{array}\right)$$

(12) $$C_{b}^{n}=\left(\begin{array}{ccc} \left(q_{0}{}^{2}+q_{1}{}^{2}-q_{2}{}^{2}-q_{3}{}^{2}\right) & 2(q_{1}q_{2}-q_{0}q_{3}) & 2(q_{1}q_{3}+q_{0}q_{2})\\ 2(q_{1}q_{2}+q_{0}q_{3}) & \left(q_{0}{}^{2}-q_{1}{}^{2}+q_{2}{}^{2}-q_{3}{}^{2}\right) & 2(q_{2}q_{3}-q_{0}q_{1})\\ 2(q_{1}q_{3}-q_{0}q_{2}) & 2(q_{2}q_{3}+q_{0}q_{1}) & \left(q_{0}{}^{2}-q_{1}{}^{2}-q_{2}{}^{2}+q_{3}{}^{2}\right) \end{array}\right)$$

The attitude angle equation based on quaternions is:

(13) $$\left(\begin{array}{c} \theta \\ \phi \\ \psi \end{array}\right)(q)=\left(\begin{array}{c} \arcsin(-2(q_{1}q_{3}-q_{0}q_{2}))\\ \mathrm{atan}2(2(q_{2}q_{3}+q_{0}q_{1}),(q_{0}{}^{2}-q_{1}{}^{2}-q_{2}{}^{2}+q_{3}{}^{2}))\\ \mathrm{atan}2(2(q_{1}q_{2}+q_{0}q_{3}),(q_{0}{}^{2}+q_{1}{}^{2}-q_{2}{}^{2}-q_{3}{}^{2})) \end{array}\right)$$

When using the extended Kalman filter for the attitude angle calculations, the measurements of the MEMS gyroscope were used to update the equation of the state, and interference-free magnetic data obtained through 1D CNN classification were used as the observation to correct errors in the one-step prediction of the equation of state. Hence, the prediction and measurement models f and h can be defined as differentiable functions of X and μ with zero mean multivariate Gaussian process and measurement noise with covariances $Q_{k}$ and $R_{k}$.

(14) $$X_{k}=f(X_{k-1},u)+N(0,Q_{k})$$

(15) $$Z=h(X_{k})+N(0,R_{k})$$

Gyroscope measurements contain errors, and though these errors only have a small effect on state predictions over short periods of time, they can accumulate over time. Indoor geomagnetic field data can be disturbed for short periods of time in some areas, but interference-free geomagnetic field data can accurately calculate heading angles of information at any time. The extended Kalman filter can combine the features of both, using interference-free magnetic data to correct for deviations in the heading angle, resulting in greater accuracy of the attitude angle. In order to reduce the amount of computation and ensure accuracy, we use quaternion to update the attitude angle. The gyroscope has zero bias instability, we also estimate the zero bias of the gyroscope in real time. Based on the above information, we can establish the state vector X:

(16) $$X={\left[\begin{array}{ccccccc} q_{0} & q_{1} & q_{2} & q_{3} & \omega_{xb} & \omega_{yb} & \omega_{zb} \end{array}\right]}^{T}$$

where $\omega_{xb}$, $\omega_{yb}$, and $\omega_{zb}$ are the three gyroscope biases.

One-step prediction of the quaternion was calculated using the derivative of quaternion:

(17) $$q_{k}=q_{k-1}+\dot{q}\cdot dt$$

The quaternion differential equation is

(18) $$\dot{q}=\frac{1}{2}q⊗\omega =\left[\begin{array}{cccc} 0 & -\omega_{x} & -\omega_{y} & -\omega_{z}\\ \omega_{x} & 0 & \omega_{z} & -\omega_{y}\\ \omega_{y} & -\omega_{z} & 0 & \omega_{x}\\ \omega_{z} & \omega_{y} & -\omega_{x} & 0 \end{array}\right]\times \left[\begin{array}{c} q_{0}\\ q_{1}\\ q_{2}\\ q_{3} \end{array}\right]$$

where ω is calculated using

(19) $$\{\begin{array}{c} \omega_{x}={\tilde{\omega }}_{x}-\omega_{xb}+\Delta \omega_{x}\\ \omega_{y}={\tilde{\omega }}_{y}-\omega_{yb}+\Delta \omega_{y}\\ \omega_{z}={\tilde{\omega }}_{z}-\omega_{zb}+\Delta \omega_{z} \end{array}$$

${\tilde{\omega }}_{x}$, ${\tilde{\omega }}_{y}$, and ${\tilde{\omega }}_{z}$ are the measurements of the MEMS gyroscope, and the gyroscope biases were unchanged during the prediction step. The equation of state based on X can be built as

(20) $$X_{k}=f(X_{k-1},\mu_{k})=\left[\begin{array}{c} q_{0}+\frac{dt}{2}*(-q_{1}(\omega_{x}-\omega_{xb})-q_{2}(\omega_{y}-\omega_{yb})-q_{3}(\omega_{z}-\omega_{zb}))\\ q_{1}+\frac{dt}{2}*(q_{0}(\omega_{x}-\omega_{xb})+q_{3}(\omega_{y}-\omega_{yb})-q_{2}(\omega_{z}-\omega_{zb}))\\ q_{2}+\frac{dt}{2}*(-q_{3}(\omega_{x}-\omega_{xb})+q_{0}(\omega_{y}-\omega_{yb})+q_{1}(\omega_{z}-\omega_{zb}))\\ q_{3}+\frac{dt}{2}*(q_{2}(\omega_{x}-\omega_{xb})-q_{1}(\omega_{y}-\omega_{yb})+q_{0}(\omega_{z}-\omega_{zb}))\\ \omega_{xb}\\ \omega_{yb}\\ \omega_{zb} \end{array}\right]$$

The Jacobian can be calculated to produce the state-transition matrix Φ:

(21) $$\Phi =\frac{\partial f}{\partial X}=\left[\begin{array}{ccccccc} 1 & -\frac{dt}{2}(\omega_{x}-\omega_{xb}) & -\frac{dt}{2}(\omega_{y}-\omega_{yb}) & -\frac{dt}{2}(\omega_{z}-\omega_{zb}) & \frac{dt}{2}q_{1} & \frac{dt}{2}q_{2} & \frac{dt}{2}q_{3}\\ \frac{dt}{2}(\omega_{x}-\omega_{xb}) & 1 & -\frac{dt}{2}(\omega_{z}-\omega_{zb}) & \frac{dt}{2}(\omega_{y}-\omega_{yb}) & -\frac{dt}{2}q_{0} & -\frac{dt}{2}q_{3} & \frac{dt}{2}q_{2}\\ \frac{dt}{2}(\omega_{y}-\omega_{yb}) & \frac{dt}{2}(\omega_{z}-\omega_{zb}) & 1 & -\frac{dt}{2}(\omega_{x}-\omega_{xb}) & \frac{dt}{2}q_{3} & -\frac{dt}{2}q_{0} & -\frac{dt}{2}q_{1}\\ \frac{dt}{2}(\omega_{z}-\omega_{zb}) & -\frac{dt}{2}(\omega_{y}-\omega_{yb}) & \frac{dt}{2}(\omega_{x}-\omega_{xb}) & 1 & -\frac{dt}{2}q_{2} & \frac{dt}{2}q_{1} & -\frac{dt}{2}q_{0}\\ 0 & 0 & 0 & 0 & 1 & 0 & 0\\ 0 & 0 & 0 & 0 & 0 & 1 & 0\\ 0 & 0 & 0 & 0 & 0 & 0 & 1 \end{array}\right]$$

The calibration algorithm proposed in this paper corrects the heading angle deviation in pedestrian inertial navigation using the geomagnetic field information obtained by 1D CNN screening without interference from the indoor environment. As the heading angle obtained from the geomagnetic signal is an absolute angle, which is not related to time, it can compensate for the phenomenon of divergence of the heading angle error with time in inertial navigation. Then, the observation vector and measurement equation of the extended Kalman filter is as follows:

(22) $$Z={\left[\begin{array}{ccc} m_{cx} & m_{cy} & m_{cz} \end{array}\right]}^{T}$$

where $m_{cx}$, $m_{cy}$, and $m_{cz}$ indicate geomagnetic field data classified by 1D CNN.

During the initialization of pedestrian inertial navigation, we can obtain geomagnetic field data $\left[\begin{array}{ccc} m_{x0} & m_{y0} & m_{z0} \end{array}\right]$ and set the value of the initialized magnetic field vector $\left[\begin{array}{ccc} 0 & b_{my} & b_{mz} \end{array}\right]$, where $b_{my}=\sqrt{m_{x0}^{2}+m_{y0}^{2}}$ and $b_{mz}=m_{z0}$. When navigating and locating pedestrians, the quaternion can be used to obtain the attitude matrix, which records the pedestrian’s attitude angle. Using the attitude matrix and the initial magnetic field vector, we are able to deduce the value of the magnetic field at the current position.

(23) $$\begin{array}{l} \left[\begin{array}{c} m_{x}\\ m_{y}\\ m_{z} \end{array}\right]=h(x_{k})\\ \left[\begin{array}{c} m_{x}\\ m_{y}\\ m_{z} \end{array}\right]=C_{n}^{b}*\left[\begin{array}{c} 0\\ b_{my}\\ b_{mz} \end{array}\right]\\ \left[\begin{array}{c} m_{x}\\ m_{y}\\ m_{z} \end{array}\right]=\left[\begin{array}{c} 2(q_{1}q_{2}+q_{0}q_{3})b_{my}+2(q_{1}q_{3}-q_{0}q_{2})b_{mz}\\ (q_{0}{}^{2}-q_{1}{}^{2}+q_{2}{}^{2}-q_{3}{}^{2})b_{my}+2(q_{2}q_{3}+q_{0}q_{1})b_{mz}\\ 2(q_{4}q_{3}-q_{0}q_{1})b_{my}+(q_{0}{}^{2}-q_{1}{}^{2}-q_{2}{}^{2}+q_{3}{}^{2})b_{mz} \end{array}\right] \end{array}$$

The Jacobian can be calculated to produce the observation matrix H:

(24) $$H=\frac{\partial h}{\partial x}=\left[\begin{array}{ccccccc} 2q_{3}b_{my}-2q_{2}b_{mz} & 2q_{2}b_{my}+2q_{3}b_{mz} & 2q_{1}b_{my}-2q_{0}b_{mz} & 2q_{0}b_{my}+2q_{1}b_{mz} & 0 & 0 & 0\\ 2q_{0}b_{my}+2q_{1}b_{mz} & -2q_{1}b_{my}+2q_{0}b_{mz} & 2q_{2}b_{my}+2q_{3}b_{mz} & -2q_{3}b_{my}+2q_{2}b_{mz} & 0 & 0 & 0\\ -2q_{2}b_{my}+2q_{0}b_{mz} & -2q_{1}b_{my}-2q_{1}b_{mz} & 2q_{3}b_{my}-2q_{2}b_{mz} & 2q_{3}b_{my}+2q_{3}b_{mz} & 0 & 0 & 0 \end{array}\right]$$

Linearization of the non-linear state equations and observational equations can be achieved using Taylor expansion, which allows the use of a Kalman filter for attitude angle calculations. The process is as follows.

Predicted state estimate:

(25) $$X_{k}=f(X_{k-1})$$

Predicted estimate covariance:

(26) $$P=\Phi P\Phi^{T}+Q$$

Measurement residual:

(27) $$Y=Z-h(X_{k})$$

Kalman gain:

(28) $$k=PH^{T}/(HPH^{T}+R)$$

Updated state estimate:

(29) $$X=X_{k}+KY$$

Updated estimate covariance:

(30) P=(I−KH)P

We implemented an algorithm based on 1D CNN magnetic anomaly classification for simulation and testing according to the above procedure of extended Kalman filtering, and we analyzed and discussed the test results for heading angle accuracy.

## 3. Experiments and Results

### 3.1. Magnetic Anomaly Detection and Trajectory Experiments

**Figure 5:**
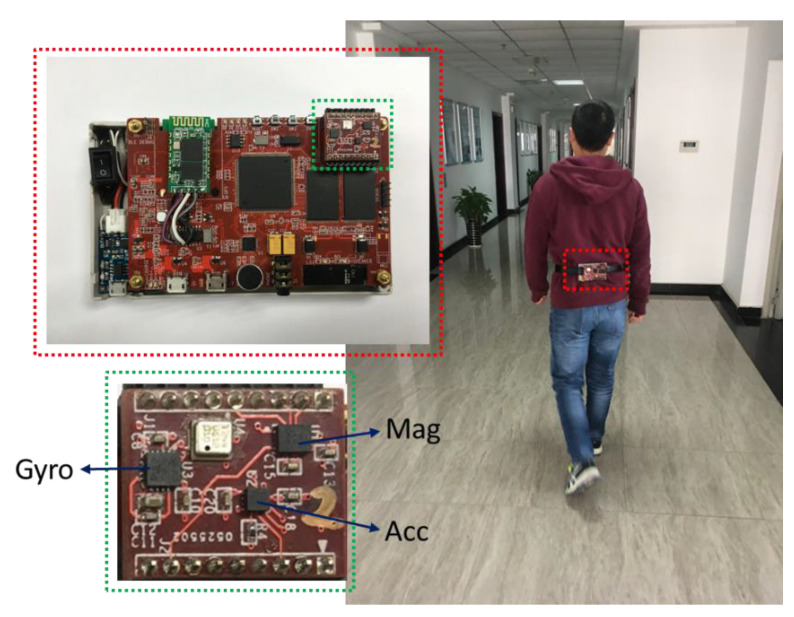
Experimental system and indoor test environment.

To verify the effectiveness of our proposed algorithm, we built a wearable navigation platform and used it to perform a series of experiments in indoor scenes. As shown in Figure 5, our system includes a MEMS accelerometer, MEMS gyroscope, magnetometer, and some auxiliary electronics component. The device is attached to the tester’s waist, and the tester walks around the room to verify the error of heading angle and the accuracy of navigation positioning accuracy. The navigation method adopts pedestrian dead reckoning based on heading angle and Weinberg’s stride estimation in Reference [30]. The test environment we chose is an office in a building. The building contains sources of magnetic field interference, such as steel structures, doors and windows, elevators, and cables, which can all contribute to generating a complex indoor magnetic environment.

**Figure 6:**
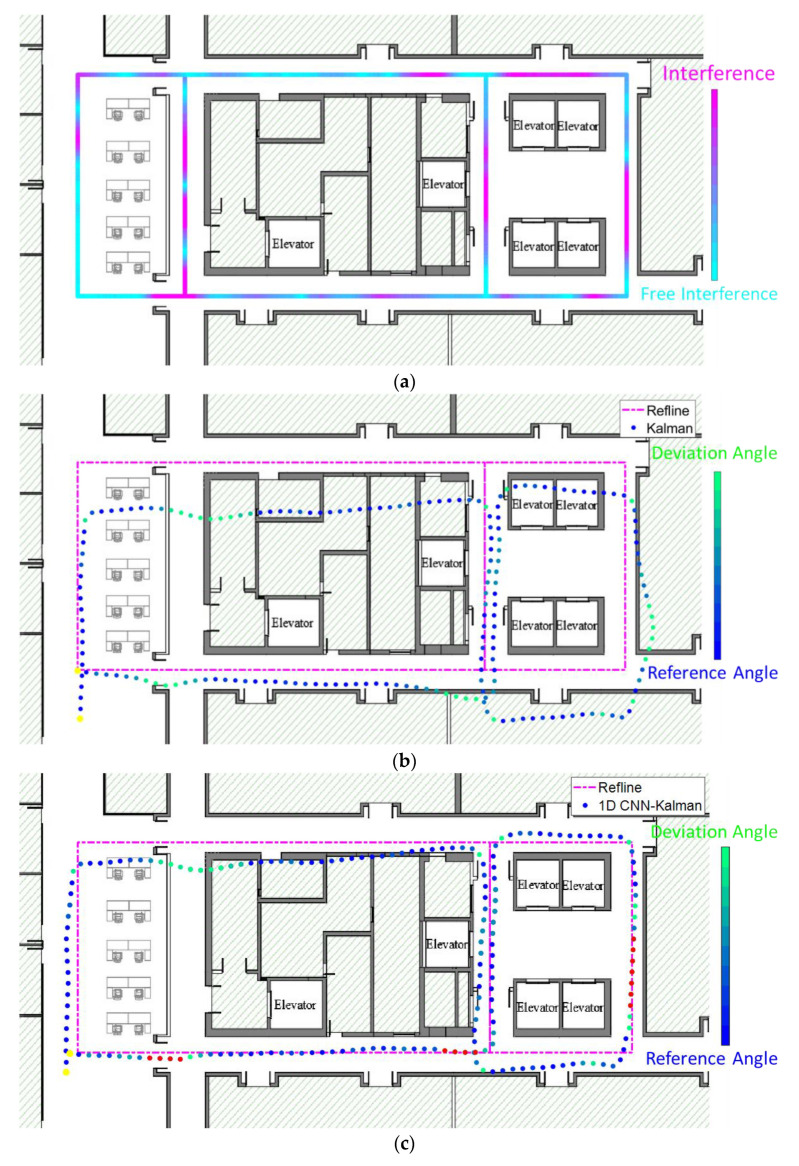
(**a**) The measurement of magnetic field in an indoor environment. (**b**) The distribution of heading angle based on Kalman. (**c**) The distribution of heading angle based on 1D CNN–Kalman.

In order to explain the working principle and performance of our algorithm, we first analyzed the magnetic field signal distribution of the indoor test track. As shown in Figure 6a, the collected magnetic field information is displayed on a plan map. The color map also indicates the magnitude of the magnetic data. We can see that the measurement values for the indoor magnetic field data are not uniform, which indicates that the indoor magnetic environment is quite complex, and geomagnetic field information has been disturbed.

Next, we studied the calculation of the heading angle in two cases, aiming to verify the effectiveness of magnetic data screening based on 1D CNN on the pedestrian inertial navigation performance of the system. In one case, we used the raw magnetic data collected by the device to directly calculate the heading. In the other case, the magnetic data are screened out by a well-trained 1D CNN before being used to calculate the heading angle. The 1D CNN discards the bad magnetic data and extracts the geomagnetic data to improve the accuracy of heading angle calculation. We plotted the trace of the tester, as shown in Figure 6b. The pink dash represents the reference trajectory, and the dash dotted line shows the navigation trajectory obtained by the heading angle which is calculated by the raw magnetic data. We also indicate the heading angle using a different color in the navigation trajectory. As can be seen, in the absence of screening using 1D CNN, the calculated heading angle deviates from the reference value. This leads to further deviations in the final navigation trajectory.

The navigation trace obtained by our algorithm with the 1D CNN screening model was plotted in Figure 6c. The heading angle is also indicated by a different color in the navigation trajectory. As can be seen, when using the measured magnetic field data, we first classify the magnetic data using the trained 1D CNN network and then calculate the heading angle for each step of the walking pedestrian. Due to the assistance in screening the magnetic data by 1D-CNN, the calculated values of the heading angle at some key corner points (marked in red) have been well corrected in Figure 6c. This shows that our algorithm significantly improves the accuracy of the system’s heading angle, thus also obtaining a more accurate trajectory.

Based on two different methods for calculating the heading angle, we can obtain two pedestrian inertial navigation trajectories as shown in Figure 6b,c. The yellow points indicate the starting and ending positions, and the positioning accuracy of the two algorithms is shown in Table 1. Using our proposed algorithm for magnetic classification by 1D CNN, we were able to reduce the localization error by 1.62 m.

### 3.2. New Comparative Experiment

**Figure 7:**
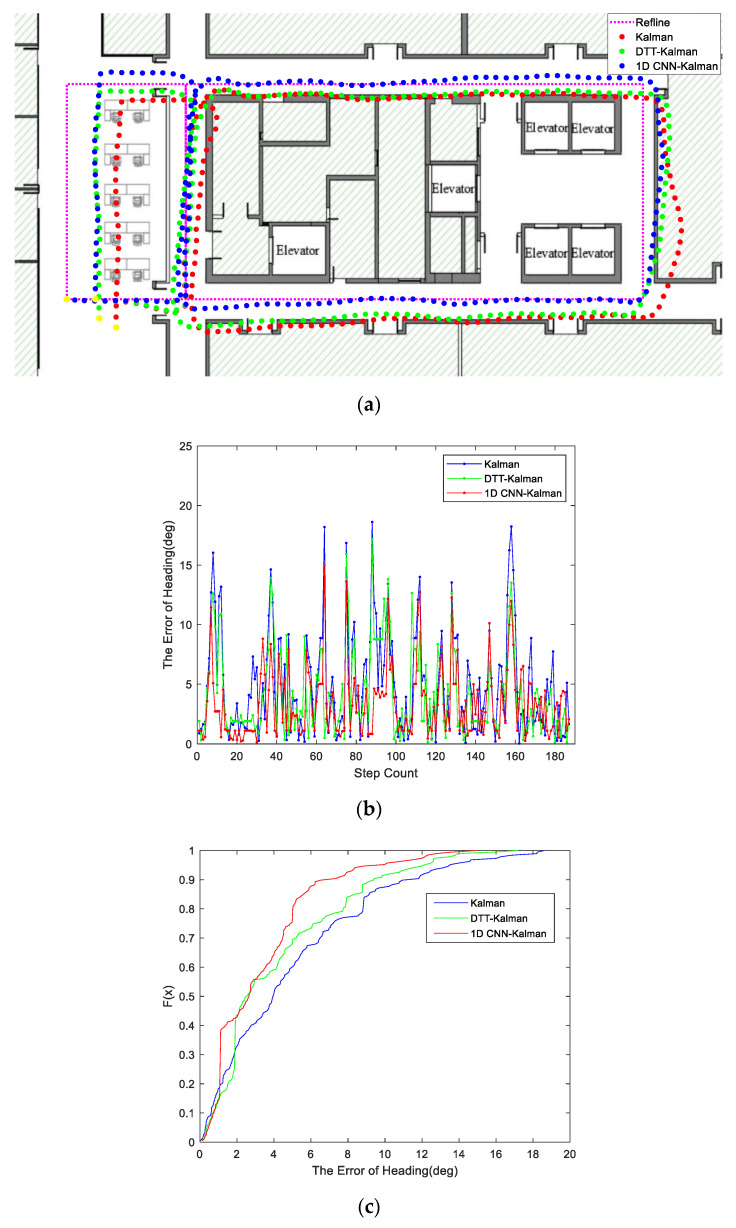
(**a**) The results of the trajectory test using three different algorithms for indoor pedestrian inertial navigation. (**b**) The error of the heading angle for the three algorithms. (**c**) The cumulative distribution function (CDF) of the heading estimation error based on the three algorithms.

For a more intuitive comparison, a new trajectory was used to verify the validity of our proposed algorithm, and we compared this algorithm with the magnetic anomaly detection algorithm (a test method based on a double threshold value, noted as the DTT method) [20]. In Figure 7a, we show navigation trajectories based on three different methods for calculating the heading angles. The pink dash represents the reference trace line of our tester. The red line was obtained from the navigation system using raw magnetic data without any screening out methods. For the green trajectory, we used the DTT method to screen the magnetic data. For calculation of the blue line, we used the proposed 1D CNN to screen the raw magnetic data. As we can see from these four lines, compared to the Kalman filtering of raw data, the DTT–Kalman screening algorithm improved the accuracy of navigation. After screening with the dual-threshold algorithm, the magnetic field data were corrected. The screened data were then applied to the calculation of the heading angle. As shown in Figure 7b, the heading angle obtained using the DTT–Kalman classification method showed some improvements in accuracy compared to the data obtained using Kalman. Further, compared with the DTT–Kalman algorithm, our proposed 1D CNN–Kalman performed better in classifying a magnetic anomaly and obtained a more accurate heading angle in Figure 7b and navigation trajectories in Figure 7a.

In Figure 7b,c, we reveal the principles behind the advantage of the 1D CNN–Kalman method with respect to the heading angle error. As can be seen from Figure 7b, the heading angle error obtained by DTT–Kalman was significantly higher than that of the 1D CNN–Kalman algorithm during the entire navigation process. This means that more accurate trajectories can be obtained using our proposed algorithm. As illustrated in Figure 7b, we traced the accommodation of the heading angle obtained by the three algorithms in the new test trajectory. From the curve of three accommodations of the heading angle, we can see that the algorithm we proposed can better distinguish magnetic anomalies, reduce the deviation of the heading angle, and thus improve the positioning accuracy of pedestrian inertial navigation. In Table 2, we list the performances of the three methods. As shown by this table, the 1D CNN–Kalman magnetic anomaly classification method results in a 30% reduction in pedestrian inertial navigation error compared to the DTT–Kalman method.

## 4. Conclusions

This paper proposes an indoor pedestrian navigation algorithm that can significantly improve navigation accuracy using magnetic data. Based on the CNN network, we designed an unsupervised learning model and obtained an algorithm that can screen out anomalies in indoor magnetic data from the measured data. In addition, by using real magnetic data obtained through screening combined with the characteristics of the gyro error mainly derived from the accumulated errors, we proposed a novel heading angle calculating method which is based on an extended Kalman filter. This method can use real indoor magnetic data to correct the accumulated errors of the inertial navigation system, thereby achieving higher accuracy. Our proposed algorithm demonstrated good adaptability and effectiveness in indoor environments when analyzed using trajectory tests in different environments. Our research provides an effective solution that addresses the issues encountered in pedestrian navigation based on wearable equipment.

## Figures and Tables

**Table 1:** Comparison of the end point positioning error in an indoor track ^1^.

Method	Kalman	1D CNN–Kalman
**Error**	2.68 m	1.06 m

^1^ Note: The track is 33.3 m long and 11.9 m wide, with a total length of 90.4 m.

**Table 2:** Comparison of the end point positioning errors in the indoor track ^2^.

Method	Kalman	DTT–Kalman	CNN–Kalman
**Error**	2.85 m	1.75 m	1.21 m

^2^ Note: The track is 33.3 m long and 11.9 m wide, with a total length of 90.4 m.
